# Effects of Oral Semaglutide on Dietary Intake and Body Composition in Japanese People With Type 2 Diabetes: A Prospective Observational Study in Clinical Practice

**DOI:** 10.1002/edm2.70295

**Published:** 2026-08-04

**Authors:** Mika Sawada, Naoko Nakanishi, Masakazu Aihara, Kanako Ohkuma, Shoko Hamano, Yoshitaka Sakurai, Rie Sekine, Satoshi Usami, Toshimasa Yamauchi, Naoto Kubota

**Affiliations:** ^1^ Department of Clinical Nutrition Therapy The University of Tokyo Tokyo Japan; ^2^ Department of Diabetes and Metabolic Diseases, Graduate School of Medicine The University of Tokyo Tokyo Japan; ^3^ Graduate School of Education The University of Tokyo Tokyo Japan; ^4^ Department of Metabolic Medicine, Faculty of Life Science Kumamoto University Kumamoto Japan

**Keywords:** body composition, food intake, glucagon‐like peptide‐1 receptor agonists (GLP‐1 RAs)

## Abstract

**Introduction:**

Glucagon‐like peptide‐1 receptor agonists are effective glucose‐lowering agents with established benefits on body weight and cardiometabolic outcomes. However, their precise effects on body composition and dietary intake patterns, including food group consumption and macronutrient energy distribution, particularly in clinical settings, remain incompletely understood.

**Methods:**

In this prospective observational study, we enrolled Japanese adults with Type 2 diabetes who were newly initiated on oral semaglutide treatment. Dietary intake patterns were assessed using a food frequency questionnaire, and body composition was assessed by bioelectrical impedance analysis. Handgrip strength and laboratory parameters were also evaluated. All parameters were assessed at both baseline (before the start of semaglutide treatment) and at a 3‐month follow‐up visit.

**Results:**

A total of 25 participants were included in the final analysis. After 3 months of oral semaglutide treatment, significant reductions in HbA1c levels and body weight were noted. Significant reductions in the percent body fat and significant increases in the percent muscle mass were also observed. No significant change was observed in the handgrip strength. While the total energy intake decreased significantly after 3 months of semaglutide treatment, the macronutrient energy distribution remained unchanged. Analysis by food groups revealed significant reductions in the intakes of fats and oils, seasonings and spices, and sweets and snacks. Notably, the daily salt intake also decreased significantly from 8.9 to 7.0 g/day.

**Conclusions:**

Oral semaglutide treatment was associated with reductions in the total energy intake and percent body fat, without significant changes in the macronutrient energy distribution or handgrip strength. Salt intake was also significantly reduced, suggesting potential clinical benefits of this treatment in the management of diabetes and related comorbidities.

## Graphical abstract

This prospective observational study evaluated changes in dietary patterns and body composition after 3 months of oral semaglutide treatment in people with Type 2 diabetes. Oral semaglutide was associated with reduced energy intake, salt intake and body fat while preserving handgrip strength.

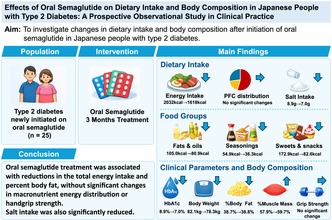

## 1 Introduction

Overweight and obesity are major risk factors for the development of Type 2 diabetes (T2D) [1]. They are central components of the cardiometabolic–kidney–metabolic (CKM) syndrome, a recently conceptualised framework that underscores the integrated pathophysiology linking adiposity, metabolic dysfunction, cardiovascular disease and chronic kidney disease [2]. Excess adipose tissue, particularly visceral fat, contributes to chronic inflammation and insulin resistance, fostering a cluster of interrelated disorders including hypertension, dyslipidemia, metabolic dysfunction‐associated steatotic liver disease (MASLD) and endothelial dysfunction [2, 3]. These pathologies synergistically elevate the risk of atherosclerotic cardiovascular disease and kidney injury in people with T2D [2]. Given this multidimensional risk, achieving and maintaining optimal body weight has become a cornerstone of modern T2D management, not only for glycemic control, but also for mitigating the broader cardiometabolic and renal sequelae associated with CKM syndrome.

Lifestyle interventions centered on medical nutritional therapy, increased physical activity, and behavioural patterns remain the foundation of treatment for people with T2D [4]. Nevertheless, long‐term adherence to these interventions is often suboptimal in clinical practice due to physiological, psychological and environmental barriers. Under these circumstances, pharmacological agents with proven weight‐lowering efficacy have emerged as critical adjuncts. Glucagon‐like peptide‐1 receptor agonists (GLP‐1 RAs) and sodium–glucose cotransporter 2 (SGLT2) inhibitors have demonstrated significant benefits for glycemic control, weight reduction, and cardiovascular and renal protection, addressing the core components of CKM syndrome [5, 6].

GLP‐1 is an incretin hormone secreted by the intestinal L‐cells in response to nutrient ingestion [7]. Its receptors are widely expressed, including in the hypothalamus, where GLP‐1 signalling plays a crucial role in central appetite regulation [7]. GLP‐1 RAs exert potent anorexigenic effects through a combination of delayed gastric emptying, reduced gastrointestinal motility, and modulation of central satiety pathways [8], which collectively lead to decreased energy intake and clinically meaningful weight loss. However, changes in body composition, including changes in fat mass and muscle mass, are also clinically important in people with T2D, particularly in the context of preventing sarcopenia and improving physical function. Along with the aforementioned anorexigenic effects, GLP‐1 RAs also improve glycemic control via glucose‐dependent stimulation of insulin secretion and suppression of glucagon secretion, with a low intrinsic potential for causing hypoglycemia [9]. Importantly, these agents have also been shown to reduce major adverse cardiovascular events and slow the progression of diabetic kidney disease, underscoring their relevance in the holistic management of T2D within the CKM paradigm [5, 10, 11].

In Japan, oral semaglutide (Rybelsus), the first non‐injectable formulation of GLP‐1 RAs, was approved in 2020 [12], offering an alternative for individuals who were unable or unwilling to use injectable therapies [13]. Despite its widespread use, the precise impact of GLP‐1 RAs, particularly oral GLP‐1 RA, on the dietary intake patterns in people with T2D remains insufficiently understood. Specifically, it is unclear how pharmacologically induced appetite suppression can alter energy intake, nutrient composition, or intakes of food groups. These effects may be further modulated by cultural factors, such as traditional dietary practices and food availability. The present study was aimed at evaluating changes in dietary intake patterns, including energy intake, macronutrient energy distribution and food group consumption, as well as body composition after the initiation of oral semaglutide treatment in Japanese people with T2D.

## 2 Materials and Methods

### 2.1 Study Design and Participants

This study was a prospective observational study conducted in the Department of Diabetes and Metabolic Medicine at the University of Tokyo Hospital. Participants were inpatients with T2D who were admitted between November 2021 and April 2023 and were newly initiated on oral semaglutide (Rybelsus) treatment during hospitalisation for medication adjustment. Eligibility criteria included adults aged 20 years or older who were suitable candidates for initiation of oral semaglutide treatment and were able to provide written informed consent. All eligible patients during this period were consecutively enrolled without investigator selection bias. The study exclusion criteria were: (1) current use of any GLP‐1 RAs; (2) prior use of oral semaglutide; (3) presence of significant gastrointestinal disease; (4) recent participation in structured weight loss interventions; or (5) diagnosis of dementia. This study was conducted in accordance with the principles of the Declaration of Helsinki with the approval of the Research Ethics Committee of the University of Tokyo Hospital (Approval No. 2021245NI). Written informed consent was obtained from all the participants prior to enrollment.

### 2.2 Sample Size Estimation

The sample size was estimated a priori for the primary outcome, namely, the change in total daily energy intake. Gibbons et al. reported a reduction in total daily energy intake of approximately 1218 kcal (5096 kJ) in people with T2D receiving oral semaglutide treatment [14], suggesting a substantial treatment effect. Because the effects observed in small, tightly controlled trials are likely to be attenuated under real‐world conditions, we conservatively assumed a moderate effect size (dz = 0.6). With a two‐sided significance level of 0.05, a statistical power of 0.80, and a paired *t*‐test, the required sample size was determined to be at least 24 participants, and the 25 participants included in the final analysis met this requirement.

### 2.3 Procedure

Oral semaglutide was administered according to standard clinical practice, beginning with a dose of 3 mg once daily, with dose escalation to 7 mg or 14 mg, as necessary, based on the tolerability and clinical response. Dietary prescriptions, including the daily energy intake and macronutrient composition, were determined by the attending physicians in accordance with the recommendations of the Japan Diabetes Society, based on the target body weight, physical activity level, age and body mass index. All the participants received individualised nutritional counselling from registered dietitians certified as diabetes educators for Japan, as medical nutritional therapy for T2D. Meal plans were individualised based on personal factors such as social background, dietary environment and cooking skills.

### 2.4 Physical, Laboratory and Activity Assessments

The participants' weight, height and body composition were measured using a direct segmental multifrequency bioelectrical impedance analyser (InBody 720; InBody Japan Inc.) [15]. Body fat mass and muscle mass (whole‐body) were obtained directly from the device output, which derives body composition data from segmental impedances measured at multiple frequencies and not through the use of a separate population‐specific prediction equation. Appendicular skeletal muscle mass (ASM) was calculated as the sum of the lean mass of the four limbs (both arms and both legs) obtained from segmental impedance analysis, and the skeletal muscle mass index (SMI) was calculated as ASM divided by height in metres squared (kg/m^2^). BMI was calculated as weight in kilograms divided by height in metres squared (kg/m^2^). Percent body fat and percent muscle mass were calculated as body fat mass and muscle mass, respectively, divided by body weight measured at the same time point and multiplied by 100. Handgrip strength was measured in both hands using a digital Smedley‐type handgrip dynamometer (Grip‐D, Sanka Co. Ltd., Japan). Two measurements were obtained for each hand and the average of the higher values from the two hands was used as an indicator of handgrip strength. Blood samples were analysed for HbA1c and blood glucose, and serum samples were analysed for LDL cholesterol, HDL cholesterol and triglycerides. Urine samples were analysed for urinary albumin and creatinine levels. The daily step count was assessed using a self‐reported five‐category questionnaire (< 3000, 3000–6000, 6000–8000, 8000–10,000 and > 10,000 steps/day) at baseline and after 3 months of oral semaglutide treatment. These measurements were performed on the first or second day of hospitalisation and repeated at the outpatient visit 3 months later.

### 2.5 Dietary Assessment

Dietary intake patterns were assessed using the Food Frequency Questionnaire Based on Food Groups (FFQg, Excel Eiyokun Ver.9, Kenpakusha) [16]. This standardised interview method was administered by registered dietitians. The FFQg allows estimation of habitual nutrient and food group intakes based on recall by the participants of their typical weekly dietary patterns over the previous 1–2 months. Nutrient intake was calculated based on portion sizes (relative to the standard amount) and the frequency of intake of various food groups, allowing for assessment of the energy, macronutrient and micronutrient intakes. Dietary assessments were performed on the second day of hospitalisation and repeated at the outpatient visit 3 months later.

### 2.6 Statistical Analysis

Descriptive statistics are expressed as means ± standard error (SE) for continuous variables and as the number of cases (proportion) for categorical variables. The normality of continuous variables was assessed using the Shapiro–Wilk test. Paired comparisons of the results recorded at baseline and at the 3‐month outpatient visit for clinical parameters, body composition, and dietary intakes were performed using paired *t*‐tests for normally distributed variables and the Wilcoxon signed‐rank test for non‐normally distributed variables. Changes in the daily step count categories between baseline and the end of 3 months of oral semaglutide treatment were compared using the Wilcoxon signed‐rank test. Sex‐specific analyses of body composition and dietary intake were performed using the same statistical methods. For paired categorical variables, including changes in antidiabetic medications, McNemar's test was used. Effect sizes for continuous variables were calculated using Glass's Δ, defined as the mean difference between baseline and follow‐up divided by the standard deviation at baseline. *p* < 0.05 was considered indicative of a statistically significant difference. All statistical analyses were conducted using SPSS, version 28.0 (IBM Corp., Armonk, NY, USA).

## 3 Results

### 3.1 Enrollment and Baseline Characteristics

**FIGURE 1:**
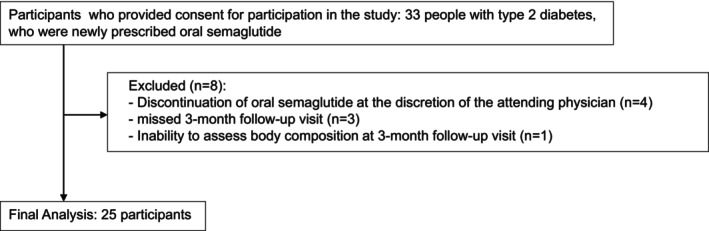
Flow diagram of participant enrollment and analysis. Flow diagram illustrating participant enrollment, inclusions, exclusions and analysis sets. Of the patients initially screened, 25 people with Type 2 diabetes who were newly initiated on oral semaglutide therapy and completed the 3‐month follow‐up examination were included in the final analysis.

A total of 33 people with T2D provided informed consent for participation in the study. Among these, 8 participants were excluded from the analysis due to physician‐directed discontinuation of oral semaglutide (*n* = 4), missed 3‐month follow‐up visits (*n* = 3), or inability to complete body composition assessment at 3‐month follow‐up visit (*n* = 1) (Figure 1). Data from the remaining 25 participants were included in the final analysis. The average age of participants was 58.3 ± 2.4 years, and 52.0% of participants were female. The average duration of diabetes was 8.0 ± 1.8 years. At baseline, 4.0% of participants had diabetic retinopathy, 40.0% had microalbuminuria, defined as a urinary albumin level of ≥ 30 mg/gCr and 60.0% had diabetic neuropathy.

### 3.2 Changes in Antidiabetic Medications After the Start of Oral Semaglutide Treatment

Table 1 shows the changes in the antidiabetic medications prescribed for the study participants after 3 months of oral semaglutide treatment. All participants were newly initiated on oral semaglutide at the dose of 3 mg taken once daily. By the 3‐month follow‐up, the majority (76.0%) remained on the 3 mg dose, while the dose had been escalated to 7 mg in 20.0% and to 14 mg in 4.0% of the study participants. Changes in antidiabetic medications other than semaglutide were also observed during the study period. DPP‐4 inhibitors, which were used by 68.0% of participants at baseline, were completely discontinued by the end of 3 months of oral semaglutide treatment (*p* < 0.05). On the other hand, the proportion of participants prescribed biguanides increased from 40% (*n* = 10) at baseline to 56% (*n* = 14) at the end of 3 months of oral semaglutide treatment, and the proportion of participants prescribed SGLT2 inhibitors rose from 28% (*n* = 7) to 52% (*n* = 13) (both *p* < 0.05).

**TABLE 1:** Changes in the antidiabetic medications after 3 months of semaglutide treatment.

Medication	Baseline (*n* = 25)	After 3 months (*n* = 25)	*p*
Semaglutide 3 mg, *n* (%)	0 (0.0)	19 (76.0)	< 0.01**
Semaglutide 7 mg, *n* (%)	0 (0.0)	5 (20.0)	0.063
Semaglutide 14 mg, *n* (%)	0 (0.0)	1 (4.0)	1.000
Insulin, *n* (%)	2 (8.0)	1 (4.0)	0.317
Sulfonylureas, *n* (%)	3 (12.0)	0 (0.0)	< 0.01**
Glinides, *n* (%)	0 (0.0)	1 (4.0)	< 0.01**
Alpha‐glucosidase inhibitors, *n* (%)	1 (4.0)	2 (8.0)	0.317
Thiazolidinediones, *n* (%)	6 (24.0)	4 (16.0)	0.157
Biguanides, *n* (%)	10 (40.0)	14 (56.0)	< 0.05*
DPP‐4 inhibitors, *n* (%)	17 (68.0)	0 (0.0)	< 0.05*
SGLT2 inhibitors, *n* (%)	7 (28.0)	13 (52.0)	< 0.05*
Imeglimin, *n* (%)	0 (0.0)	0 (0.0)	—
No oral antidiabetic medication, *n* (%)	5 (20.0)	0 (0.0)	< 0.01**

*Note:* Values are presented as the number (proportion) of participants receiving each medication at baseline and after 3 months of semaglutide treatment. *p*‐values were calculated using McNemar's test for paired categorical variables. **p < 0.01; *p < 0.05. *p*‐values shown in bold indicate statistically significant differences.

Abbreviations: DPP‐4 inhibitor, dipeptidyl peptidase‐4 inhibitor; SGLT2 inhibitor, sodium–glucose cotransporter 2 inhibitor.

### 3.3 Clinical Parameters and Body Composition

The baseline characteristics and changes in clinical parameters and body composition in the participants after 3 months of oral semaglutide treatment are summarised in Table 2. HbA1c decreased statistically significantly from 8.9% ± 0.3% at baseline to 7.0% ± 0.2% at the end of 3 months of oral semaglutide treatment (*p* < 0.01; Glass's Δ = −1.24). Serum HDL cholesterol significantly increased, whereas no significant changes were noted in the serum LDL cholesterol or triglyceride levels after 3 months of treatment. Urinary albumin decreased significantly from 44.1 ± 11.6 mg/gCr at baseline to 21.5 ± 4.5 mg/gCr after 3 months of treatment (*p* < 0.01; Glass's Δ = −0.26).

**TABLE 2:** Changes in clinical parameters and body composition after 3 months of oral semaglutide treatment.

Variables	Baseline (*n* = 25)	After 3 months (*n* = 25)	*p*	Effect size
**Clinical parameters**				
HbA1c (%)^a^	8.9 ± 0.3	7.0 ± 0.2	< 0.01**	−1.24
Fasting plasma glucose (mg/dL)^b^	182.5 ± 19.1	139.2 ± 8.4	0.451	−0.45
Serum LDL cholesterol (mg/dL)^a^	107.0 ± 6.6	103.3 ± 6.9	0.752	−0.07
Serum HDL cholesterol (mg/dL)^a^	51.2 ± 3.5	57.8 ± 3.3	< 0.05*	0.31
Serum triglycerides (mg/dL)^a^	137.7 ± 11.6	138.9 ± 19.1	0.790	0.08
Urinary albumin (mg/gCr)^b^	44.1 ± 11.6	21.5 ± 4.5	< 0.01**	−0.26
**Body composition**				
Weight (kg)^b^	82.1 ± 4.1	78.3 ± 4.0	< 0.01**	−0.19
BMI (kg/m^2^)^b^	30.9 ± 1.5	29.5 ± 1.5	< 0.01**	−0.18
Body fat (kg)^a^	32.9 ± 3.2	30.0 ± 3.1	< 0.01**	−0.18
Percent body fat (%)^a^	38.7 ± 2.3	36.8 ± 2.2	< 0.01**	−0.16
Muscle mass (kg)^a^	46.5 ± 2.0	45.6 ± 1.8	0.053	−0.10
Percent muscle mass (%)^a^	57.9 ± 2.2	59.7 ± 2.1	< 0.01**	0.16
ASM (kg)^b^	21.5 ± 1.1	20.5 ± 1.0	< 0.01**	−0.18
SMI (kg/m^2^)^a^	8.0 ± 0.3	7.6 ± 0.3	< 0.05*	−0.22
Handgrip strength (kg)^a^	27.7 ± 1.9	28.4 ± 1.8	0.338	0.08

*Note:* Values are presented as means ± SE. *p*‐values are calculated using paired *t*‐tests for normally distributed variables and the Wilcoxon signed‐rank test for non‐normally distributed variables. Effect size denotes Glass's Δ (mean difference between baseline and follow‐up divided by the standard deviation at baseline). **p < 0.01; *p < 0.05. *p*‐values shown in bold indicate statistically significant differences.

Abbreviations: ASM, appendicular skeletal muscle mass; BMI, body mass index; HbA1c, haemoglobin A1c; HDL, high‐density lipoprotein; LDL, low‐density lipoprotein; SMI, skeletal muscle mass index.

^a^ Paired *t*‐test.

^b^ Wilcoxon's signed‐rank test.

The body weight of the participants decreased significantly from 82.1 ± 4.1 kg at baseline to 78.3 ± 4.0 kg after 3 months of oral semaglutide treatment (*p <* 0.01; Glass's Δ = −0.19); corresponding decrease of the BMI was also observed (*p <* 0.01; Glass's Δ = −0.18). Significant decreases of both the body fat mass and percent body fat were observed (*p <* 0.01; Glass's Δ = −0.18 and −0.16, respectively). On the other hand, while the muscle mass showed a trend towards decrease from 46.5 ± 2.0 kg at baseline to 45.6 ± 1.8 kg after 3 months of treatment (*p* = 0.053; Glass's Δ = −0.10), the percent muscle mass significantly increased from 57.9% ± 2.2% at baseline to 59.7% ± 2.1% after 3 months of treatment (*p <* 0.01; Glass's Δ = 0.16). The ASM and SMI also decreased significantly after 3 months of oral semaglutide treatment (*p* < 0.01 and *p* < 0.05, respectively). No significant change in the handgrip strength was observed after 3 months of oral semaglutide treatment.

The daily step count category did not change significantly between baseline and the end of 3 months of oral semaglutide treatment (Tables S1 and S2). At the individual level, the step count category remained unchanged in 19 participants (76.0%), decreased in 4 participants (16.0%), and increased in 2 participants (8.0%).

### 3.4 Dietary Intake

**FIGURE 2:**
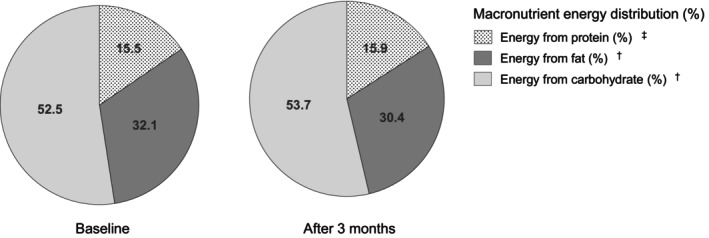
Changes in the macronutrient energy distribution after 3 months of oral semaglutide treatment. Macronutrient energy distribution (%) at baseline and after 3 months of oral semaglutide treatment, showing the relative contributions of proteins, fats and carbohydrates to the total energy intake. Data are presented as means. Paired comparisons between baseline and after 3 months of treatment were performed using paired *t*‐tests or the Wilcoxon signed‐rank test, as appropriate. ^‡^Wilcoxon's signed‐rank test; ^†^paired *t*‐test. No statistically significant differences were observed.

Changes in the dietary intake patterns of the participants after 3 months of oral semaglutide treatment are summarised in Table 3. Total energy intake decreased significantly from 2032.5 ± 152.4 kcal/day at baseline to 1618.2 ± 90.0 kcal/day after 3 months of semaglutide treatment (*p <* 0.01; Glass's Δ = −0.54). Significant decreases in the intakes of carbohydrates, proteins and fats were also noted. However, no significant changes were observed in the macronutrient composition of the diet (carbohydrates: 52.5% ± 1.3% vs. 53.7% ± 1.3%; proteins: 15.5% ± 0.5% vs. 15.9% ± 0.4%; fats: 32.1% ± 0.9% vs. 30.4% ± 1.0%) (Figure 2). The participants showed a significant decrease in the salt intake from 8.9 ± 0.5 g/day at baseline to 7.0 ± 0.5 g/day after 3 months of semaglutide treatment (*p* < 0.01), with a relatively large effect size (Glass's Δ = −0.71). Analysis by food groups revealed significant reductions in the intakes of fats and oils, seasonings and spices and sweets and snacks (*p* < 0.01; Glass's Δ = −0.48, −0.66 and −0.51, respectively). In contrast, no significant changes were observed in the intakes of cereals, potatoes, fruits, or soy and soy products, fish and shellfish, meat, eggs, milk and dairy products, nuts and seeds, vegetables, seaweed, sugars and sweeteners, alcohol, or non‐alcoholic beverages.

**TABLE 3:** Changes in dietary intakes after 3 months of oral semaglutide treatment.

Variable	Baseline (*n* = 25)	After 3 months (*n* = 25)	*p*	Effect size
**Nutrient**				
Energy (kcal/day)^b^	2032.5 ± 152.4	1618.2 ± 90.0	< 0.01**	−0.54
Carbohydrate (g/day)^b^	250.9 ± 23.6	203.6 ± 12.6	< 0.01**	−0.40
Protein (g/day)^a^	76.6 ± 4.6	63.6 ± 3.1	< 0.01**	−0.57
Fat (g/day)^b^	71.4 ± 4.8	54.9 ± 3.6	< 0.01**	−0.69
Saturated Fatty Acids (g/day)^b^	22.4 ± 1.6	17.7 ± 1.3	< 0.01**	−0.57
Monounsaturated Fatty Acids (g/day)^b^	25.8 ± 1.9	19.6 ± 1.3	< 0.01**	−0.67
Polyunsaturated Fatty Acids (g/day)^b^	15.0 ± 1.1	11.1 ± 0.8	< 0.01**	−0.70
Cholesterol (mg/day)^a^	376.9 ± 24.2	312.9 ± 17.9	< 0.05*	−0.53
Salt (g/day)^a^	8.9 ± 0.5	7.0 ± 0.5	< 0.01**	−0.71
**Food group**				
Cereals (kcal/day)^b^	696.4 ± 78.2	643.7 ± 48.6	0.581	−0.13
Potatoes (kcal/day)^b^	26.5 ± 5.5	21.2 ± 4.3	0.422	−0.19
Fruits (kcal/day)^a^	51.4 ± 11.4	43.5 ± 8.0	0.445	−0.14
Soy and soy products (kcal/day)^b^	81.9 ± 18.3	69.2 ± 11.5	0.936	−0.14
Fish and shellfish (kcal/day)^a^	92.6 ± 11.0	82.8 ± 9.2	0.483	−0.18
Meat (kcal/day)^b^	264.6 ± 37.6	210.5 ± 23.3	0.153	−0.29
Eggs (kcal/day)^b^	63.4 ± 7.3	55.2 ± 5.5	0.268	−0.22
Milk and dairy products (kcal/day)^a^	167.3 ± 24.3	127.5 ± 16.7	0.108	−0.33
Nuts and seeds (kcal/day)^b^	40.9 ± 17.9	21.8 ± 6.6	0.881	−0.21
Fats and oils (kcal/day)^b^	105.0 ± 10.1	80.9 ± 8.5	< 0.01**	−0.48
Green and yellow vegetables (kcal/day)^a^	20.5 ± 3.2	14.7 ± 1.7	0.288	−0.37
Other vegetables (kcal/day)^a^	52.7 ± 5.2	42.9 ± 4.6	0.082	−0.38
Seaweed (kcal/day)^a^	4.1 ± 0.9	3.4 ± 0.6	0.397	−0.17
Sugars and sweeteners (kcal/day)^a^	23.0 ± 4.3	17.1 ± 4.2	0.261	−0.27
Seasonings and spices (kcal/day)^a^	54.9 ± 5.6	36.3 ± 5.8	< 0.01**	−0.66
Alcohol (kcal/day)^b^	78.5 ± 35.7	48.9 ± 16.8	0.386	−0.17
Non‐alcoholic beverages (kcal/day)^b^	35.9 ± 16.3	16.0 ± 8.8	0.534	−0.24
Sweets and Snacks (kcal/day)^b^	172.9 ± 35.5	82.6 ± 17.3	< 0.01**	−0.51

*Note:* Values are presented as means ± SE. *p*‐values are calculated using paired *t*‐tests for normally distributed variables and the Wilcoxon signed‐rank test for non‐normally distributed variables. Effect size denotes Glass's Δ (mean difference between baseline and follow‐up divided by the standard deviation at baseline). **p < 0.01; *p < 0.05. *p*‐values shown in bold indicate statistically significant differences.

^a^ Paired *t*‐test.

^b^ Wilcoxon's signed‐rank test.

### 3.5 Gender‐Specific Analysis

Gender‐specific analyses of the body composition and dietary intake patterns are summarised in Table 4. Both the male and female participants showed significant decreases in body weight and BMI after 3 months of oral semaglutide treatment (*p* < 0.05). Female participants showed a significant decrease in body fat mass, whereas in male participants, the decrease in body fat mass was not statistically significant. In female participants, while the muscle mass, ASM and SMI decreased significantly, the percent muscle mass showed a significant increase. No significant changes in muscle mass, percent muscle mass, ASM, or SMI were observed in male participants. No significant changes in handgrip strength were observed in either male or female participants.

**TABLE 4:** Changes in body composition and dietary intakes after 3 months of oral semaglutide treatment by sex.

Variable	Male (*n* = 12)				Female (*n* = 13)			
	Baseline	After 3 months	*p*	Effect size	Baseline	After 3 months	*p*	Effect size
**Body composition**								
Weight (kg)	79.4 ± 3.9	76.8 ± 4.4	< 0.05* ^a^	−0.20	84.6 ± 7.1	79.7 ± 6.7	< 0.01** ^b^	−0.19
BMI (kg/m^2^)	27.8 ± 1.4	26.9 ± 1.6	< 0.05* ^a^	−0.18	33.7 ± 2.4	31.9 ± 2.2	< 0.01** ^b^	−0.22
Body fat (kg)	25.5 ± 3.5	23.3 ± 3.4	0.092	−0.18	39.7 ± 4.7	36.2 ± 4.6	< 0.01** ^a^	−0.21
Percent body fat (%)	31.6 ± 3.3	29.6 ± 2.7	0.131	−0.17	45.3 ± 2.1	43.3 ± 2.4	0.058	−0.26
Muscle mass (kg)	50.9 ± 2.6	50.5 ± 2.3	0.486	−0.05	42.4 ± 2.6	41.0 ± 2.2	< 0.01** ^a^	−0.15
Percent muscle mass (%)	64.6 ± 3.1	66.4 ± 2.5	0.129	0.17	51.7 ± 1.9	53.4 ± 2.2	< 0.05* ^a^	0.25
ASM (kg)	23.5 ± 1.4	23.0 ± 1.2	0.154	−0.09	19.6 ± 1.5	18.2 ± 1.2	< 0.05* ^a^	−0.27
SMI (kg/m^2^)	8.13 ± 0.38	8.01 ± 0.33	0.299	−0.09	7.82 ± 0.45	7.31 ± 0.37	< 0.05* ^a^	−0.31
Handgrip strength (kg)	33.9 ± 1.5	34.6 ± 1.4	0.624	0.16	23.6 ± 2.4	24.2 ± 2.1	0.379	0.07
**Nutrient**								
Energy (kcal/day)	2106.8 ± 279.3	1667.4 ± 141.6	< 0.05* ^b^	−0.45	1963.9 ± 150.1	1572.8 ± 117.5	< 0.05* ^a^	−0.72
Carbohydrate (g/day)	253.0 ± 42.7	210.9 ± 21.4	0.308	−0.28	248.9 ± 24.3	196.9 ± 14.8	< 0.05* ^b^	−0.59
Protein (g/day)	78.6 ± 8.5	63.4 ± 4.4	0.052	−0.52	74.8 ± 4.4	63.8 ± 4.5	< 0.05* ^a^	−0.69
Fat (g/day)	75.1 ± 8.6	53.0 ± 4.8	< 0.01** ^b^	−0.74	68.0 ± 4.8	56.6 ± 5.5	< 0.05* ^a^	−0.65
Saturated Fatty Acids (g/day)	22.6 ± 2.7	16.3 ± 1.5	< 0.05* ^b^	−0.67	22.2 ± 2.0	19.0 ± 2.0	0.098	−0.44
Monounsaturated Fatty Acids (g/day)	27.9 ± 3.4	19.2 ± 1.9	< 0.01** ^b^	−0.74	23.8 ± 1.7	20.0 ± 2.0	< 0.05* ^a^	−0.64
Polyunsaturated Fatty Acids (g/day)	16.3 ± 2.2	11.1 ± 1.1	< 0.05* ^a^	−0.68	13.9 ± 0.8	11.1 ± 1.2	< 0.05* ^a^	−0.99
Cholesterol (mg/day)	384.3 ± 34.3	311.9 ± 24.0	0.107	−0.61	370.0 ± 35.3	313.9 ± 27.3	0.110	−0.44
Energy from carbohydrate (%)	51.8 ± 2.4	55.7 ± 1.9	0.175	0.48	53.1 ± 1.5	51.8 ± 1.6	0.362	−0.24
Energy from protein (%)	15.5 ± 0.9	15.5 ± 0.7	0.954	0.02	15.5 ± 0.6	16.3 ± 0.4	< 0.01** ^b^	0.39
Energy from fat (%)	32.8 ± 1.6	28.8 ± 1.3	< 0.05* ^a^	−0.74	31.4 ± 1.0	31.9 ± 1.5	0.687	0.12
Salt (g/day)	8.2 ± 0.8	6.7 ± 0.6	0.063	−0.55	9.5 ± 0.7	7.3 ± 0.8	< 0.05* ^a^	−0.90
**Food groups**								
Cereals (kcal/day)	758.0 ± 140.5	723.1 ± 83.5	0.738	−0.07	639.5 ± 79.2	570.4 ± 47.6	0.753	−0.24
Potatoes (kcal/day)	19.1 ± 4.9	16.7 ± 4.9	0.463	−0.14	33.5 ± 9.4	25.5 ± 7.0	0.646	−0.23
Fruits (kcal/day)	28.3 ± 12.9	35.5 ± 9.7	0.547	0.16	72.7 ± 16.7	50.9 ± 12.4	0.193	−0.36
Soy and soy products (kcal/day)	80.2 ± 37.0	68.0 ± 13.6	0.346	−0.10	83.5 ± 11.4	70.4 ± 18.8	0.455	−0.32
Fish and shellfish (kcal/day)	88.6 ± 15.6	80.3 ± 10.4	0.597	−0.15	96.2 ± 16.1	85.2 ± 15.3	0.638	−0.19
Meat (kcal/day)	316.1 ± 71.8	221.9 ± 39.4	0.158	−0.38	217.2 ± 26.2	200.0 ± 27.4	0.529	−0.18
Eggs (kcal/day)	65.6 ± 10.2	58.4 ± 7.4	0.545	−0.20	61.4 ± 10.8	52.3 ± 8.2	0.376	−0.23
Milk and dairy products (kcal/day)	135.4 ± 34.6	103.9 ± 23.7	0.447	−0.26	196.7 ± 33.3	149.3 ± 22.6	0.125	−0.40
Nuts and seeds (kcal/day)	47.6 ± 34.4	21.7 ± 6.7	0.678	−0.22	34.7 ± 14.9	21.9 ± 11.3	0.424	−0.24
Fats and oils (kcal/day)	123.6 ± 17.0	82.1 ± 11.7	< 0.05* ^a^	−0.71	87.9 ± 9.8	79.9 ± 12.6	0.339	−0.23
Green and yellow vegetables (kcal/day)	15.2 ± 4.9	13.7 ± 2.0	0.780	−0.09	25.4 ± 3.7	15.6 ± 2.7	0.069	−0.73
Other vegetables (kcal/day)	48.2 ± 6.9	40.7 ± 6.6	0.298	−0.31	56.8 ± 7.8	44.9 ± 6.5	0.184	−0.43
Seaweed (kcal/day)	4.2 ± 1.3	2.9 ± 1.0	0.460	−0.29	4.1 ± 1.4	3.8 ± 0.8	0.716	−0.07
Sugars and sweeteners (kcal/day)	19.6 ± 7.5	15.6 ± 5.2	0.671	−0.15	26.1 ± 4.8	18.4 ± 6.6	< 0.05* ^b^	−0.45
Seasonings and spices (kcal/day)	56.0 ± 9.0	38.5 ± 10.7	< 0.05* ^a^	−0.56	53.8 ± 7.2	34.3 ± 5.6	0.098	−0.75
Alcohol (kcal/day)	98.8 ± 42.8	92.3 ± 29.8	0.815	−0.04	59.7 ± 57.4	8.9 ± 7.7	0.285	−0.25
Non‐alcoholic beverages (kcal/day)	42.0 ± 21.8	5.1 ± 5.1	0.138	−0.49	30.2 ± 24.8	26.0 ± 16.1	0.345	−0.05
Sweets and Snacks (kcal/day)	160.3 ± 64.6	47.1 ± 18.0	< 0.01** ^b^	−0.51	184.4 ± 36.1	115.3 ± 26.2	0.136	−0.53

*Note:* Values are presented as means ± SE. *p*‐values are calculated using paired *t*‐tests for normally distributed variables and the Wilcoxon signed‐rank test for non‐normally distributed variables. Effect size denotes Glass's Δ (mean difference between baseline and follow‐up divided by the standard deviation at baseline). **p < 0.01; *p < 0.05. *p*‐values shown in bold indicate statistically significant differences.

Abbreviations: ASM, appendicular skeletal muscle mass; BMI, body mass index; SMI, skeletal muscle mass index.

^a^ Paired *t*‐test.

^b^ Wilcoxon's signed‐rank test.

In both male and female participants, a significant decrease in the total energy intake was observed after 3 months of semaglutide treatment. The female participants showed significant decreases in carbohydrate, protein, as well as fat intakes, whereas the male participants showed a relatively more marked decrease in the fat intake. Consistent with this finding, the male participants showed significant reductions in the intakes of fats and oils and sweets and snacks. Because a more pronounced reduction in sweets and snacks intake was observed in male participants, changes in intake by category after 3 months of oral semaglutide treatment were examined (Table S3). Although the specific categories that changed differed between male and female participants, intake of chewing‐required sweet foods decreased significantly in both groups.

## 4 Discussion

In this prospective observational study of Japanese people with T2D, we investigated the effects of oral semaglutide treatment on the dietary intake patterns and body composition. After 3 months of treatment, several important findings were observed. First, significant reductions in HbA1c and body weight were noted. Second, a significant decrease in the percent body fat and a significant increase in the percent muscle mass were observed, whereas the handgrip strength remained unchanged. Third, a significant decrease in the total energy intake was noted, while the macronutrient energy distribution remained unchanged. Fourth, significant reductions in the intakes of specific food groups, namely fats and oils, seasonings and spices and sweets and snacks, were observed. Finally, as a novel and noteworthy finding, the participants also showed a significant decrease in their daily salt intake.

A major clinical concern with GLP‐1 RAs induced weight loss is the potential loss of fat‐free mass, including skeletal muscle mass [17]. In the present study, the body weight decreased by 4.6% (3.8 kg) over 3 months of treatment, corresponding to an average weekly weight loss of approximately 0.39%. Previous studies examining the relationship between the rate of weight loss and changes in body composition have reported that fat‐free mass can be preserved when the rate of weight loss does not exceed approximately 1.0% per week [18]. In our participants, the percent muscle mass increased, while the ASM and SMI decreased significantly, particularly in women. The ASM and SMI more directly reflect absolute changes in muscle mass, whereas the percent muscle mass is a relative index representing the proportion of muscle mass to body weight. Therefore, these measures may show different patterns of change during weight loss. The observed increase in the percent muscle mass may reflect a relative change driven primarily by the greater proportional reduction in fat mass, rather than preservation of absolute muscle mass. Handgrip strength remained unchanged, although it is generally considered as an insensitive measure over such short periods as the 3‐month observation period in this study. These distinctions should be considered when interpreting the potential risk of sarcopenia during oral semaglutide treatment. Sex differences in these muscle‐related indices during semaglutide treatment warrant further investigation.

Regarding dietary composition, the macronutrient energy distribution remained unchanged after the start of oral semaglutide treatment. When the dietary intake was analysed by food groups, no significant changes were observed in the intakes of staple foods (grains), main dishes (soybeans and soybean products, fish and shellfish, eggs and meat), or side dishes (vegetables and seaweed), which constitute the core components of a balanced diet. These findings suggest that semaglutide may reduce overall food intake proportionally, rather than selectively suppressing specific nutrients or food groups important for maintaining dietary balance in individuals with T2D. In line with these observations, previous studies have reported that liraglutide reduces total energy intake without altering the macronutrient composition [19].

One of the most notable findings of the present study was the significant reduction in salt intake of the participants, from 8.9 ± 0.5 g/day at baseline to 7.0 ± 0.5 g/day at 3 months after the start of oral semaglutide treatment. This change indicates a clinically meaningful improvement in dietary salt intake. In general, populations in East Asian countries, including Japan, China and South Korea, show higher salt consumption than those in Western countries [20], frequently exceeding the World Health Organization recommendation of < 5.0 g/day [21]. Importantly, the primary dietary sources of salt also differ by region. Whereas salt intake in Western countries largely derives from processed foods, salt consumption in Asian populations is predominantly attributable to discretionary addition of salt and seasonings used in cooking, such as soy sauce and miso [20]. Consistent with these regional characteristics, the present study demonstrated a significant reduction in the intake of seasonings in the participants receiving oral semaglutide treatment, suggesting that decreased use of seasonings—an important source of dietary salt in Asian diets—may have contributed substantially to the observed reduction in salt intake. Supporting this interpretation, previous studies investigating taste perception in individuals treated with incretin‐based therapies have reported that 26.7% of semaglutide users experienced increased sensitivity to salty taste [22]. In addition, another study demonstrated a reduced craving for savoury foods during semaglutide treatment [23]. Previous research has shown that high‐salt diets can increase ad libitum energy intake [24]. Furthermore, data from the international INTERMAP study, conducted in Japan, China, the United Kingdom and the United States, indicated that even after adjustment for total energy intake, each 1 g/day increase in salt intake was associated with a 21% higher risk of overweight (BMI ≥ 25) [25]. These findings suggest that salt intake may contribute to the risk of obesity, independently of the total energy consumption. In this context, the increase in taste sensitivity to salt‐induced by GLP‐1 RAs therapy may enable individuals receiving these drugs to achieve dietary satisfaction with smaller amounts of salt. Such an effect could also attenuate salt‐induced hyperphagia, thereby facilitating reduction in overall food intake and promoting weight loss. Although GLP‐1 RA are known to enhance urinary sodium excretion [26], the present findings also raise the possibility that semaglutide may exert a salt‐reducing effect through modulation of taste perception. If confirmed, this mechanism could represent an additional pathway by which semaglutide contributes to renal protection. Further studies are warranted to elucidate this potential mechanism in greater detail.

In regard to the effect of semaglutide on the food group intake patterns, oral semaglutide treatment was associated with reductions in the intakes of fats and oils, as well as those of sweets and snacks. These findings are consistent with those of a randomised controlled trial reported by Gibbons et al., which demonstrated significant reductions in the consumption of high‐fat and sweet foods after 12 weeks of oral semaglutide administration [14]. In this study, following oral semaglutide treatment for 3 months, the study participants showed a significant decrease in the intakes of sweets and snacks that require chewing but not in the intakes of sweets and snacks that require little or no chewing. They also showed no significant changes in the intake of non‐alcoholic beverages following oral semaglutide treatment for 3 months. The rate of gastric emptying depends on the form of food. In general, emptying of solid foods occurs more slowly than that of liquid foods [27]. Because GLP‐1 RAs delay gastric emptying, this mechanism may partly account for the significant reduction in the intakes of sweets and snacks that require chewing observed in both male and female in this study. Taken together, oral semaglutide may be more effective in inducing weight loss in individuals who habitually consume foods, sweets and snacks that require chewing, whereas individuals who primarily consume items that require little or no chewing may exhibit a reduced weight loss response to the drug. Therefore, future nutrition counselling focusing on the ‘texture’ and ‘chewing characteristics’ of foods may be beneficial for patients receiving semaglutide treatment. Further studies are needed to elucidate the underlying mechanisms.

Several confounding factors should be considered when interpreting these findings. During the follow‐up period, there were substantial changes in the concomitantly prescribed antidiabetic medications: DPP‐4 inhibitors were discontinued in all participants who had been using them, whereas the proportions of individuals prescribed biguanides and SGLT2 inhibitors increased significantly. These changes could also have influenced the dietary intake and body composition. With respect to dietary intake, metformin has been shown to suppress appetite and lower caloric intake [28]. SGLT2 inhibitors induce caloric loss through glucosuria, promoting a negative energy balance. Conversely, SGLT2 inhibitors have also been reported to elicit a compensatory increase in energy intake (compensatory hyperphagia) that partly offsets this loss [29, 30, 31]. With respect to body composition, SGLT2 inhibitors reduce total body water, predominantly in the extracellular compartment [32]. Because BIA estimates lean and muscle mass from body water, fluid shifts could influence the body composition estimates; BIA is sensitive to the hydration status and differs in this respect from criterion methods such as DXA [33]. Taken together, the observed changes in dietary intake and body composition may not be entirely attributable to oral semaglutide treatment alone, and may reflect, at least in part, the combined pharmacological and fluid‐related effects of the concurrently administered medications.

**Strengths and Limitations:** This study has several strengths. First, as a prospective observational study, it simultaneously evaluated changes in body weight, body composition and dietary intake patterns after the initiation of oral semaglutide treatment. This study design allowed for an exploratory assessment of the associations between changes in dietary intake patterns and alterations in body composition over time. In addition, dietary intake was assessed using a validated food frequency questionnaire, which enabled quantitative evaluation of both changes in nutrient intakes and food group intakes.

However, several limitations of this study should be acknowledged. First, this was a single‐center study with a relatively small sample size, which may limit the generalizability of the findings; in addition, the sex‐stratified subgroup analyses were exploratory and under‐powered, and their results should be interpreted with caution. Second, the follow‐up period was relatively short (3 months), precluding assessment of the longer‐term changes in dietary behaviours and body composition. Third, dietary intake was assessed using the FFQg on the second day of hospitalisation and recall bias related to the environmental changes associated with hospitalisation cannot be entirely excluded despite our additional use of standardised interviews by registered dietitians. Fourth, body composition was assessed by BIA rather than by a criterion method such as DXA; because BIA is sensitive to the hydration status, fluid shifts associated with concurrently administered medications could have affected the estimates. Fifth, owing to the observational design of the study and absence of a control group of hospitalised individuals with diabetes who did not receive oral semaglutide, it would be impossible to exclude the independent effects of hospitalisation on the results of the study or indeed establish causal relationships based on the results of this study. Finally, because there were modifications to concomitantly prescribed antidiabetic medications in the study participants during the follow‐up period, the observed changes in dietary intake and body composition may not be entirely attributable to the oral semaglutide treatment alone, which we regard as a major limitation of this study.

## 5 Conclusion

In conclusion, oral semaglutide treatment was associated with reduced total energy intake without changes in the macronutrient energy distribution in people with T2D. The percent body fat decreased, while the percent muscle mass increased and the handgrip strength remained unchanged. In addition, salt intake was significantly reduced. These findings suggest the potential clinical benefits in the management of diabetes and related comorbidities. Further long‐term studies are needed to clarify the relationships between changes in the eating behaviours, metabolism and body composition induced by oral GLP‐1 RA treatment.

## Author Contributions

**Rie Sekine:** writing – review and editing, investigation, resources. **Naoko Nakanishi:** conceptualization, methodology, data curation, investigation, writing – review and editing. **Toshimasa Yamauchi:** writing – review and editing, supervision, resources. **Masakazu Aihara:** conceptualization, methodology, investigation, writing – review and editing. **Yoshitaka Sakurai:** investigation, writing – review and editing. **Mika Sawada:** formal analysis, methodology, data curation, investigation, funding acquisition, visualization, writing – original draft, writing – review and editing. **Kanako Ohkuma:** investigation, writing – review and editing. **Naoto Kubota:** conceptualization, methodology, supervision, resources, writing – review and editing, writing – original draft, project administration, validation. **Shoko Hamano:** investigation, writing – review and editing. **Satoshi Usami:** validation, formal analysis, supervision, writing – review and editing.

## Funding

This study was supported by a medical staff research grant from the Japan Association for Diabetes Education and Care (Grant No. 2022‐MED‐001), awarded to M.S. The funder had no role in the study design, data collection, data interpretation, or writing of the report.

## Conflicts of Interest

The authors declare no conflicts of interest.

## Supporting information


**Table S1:** Daily step count distribution at baseline and after 3 months of oral semaglutide treatment.
**Table S2:** Individual changes in the daily step count category after 3 months of oral semaglutide treatment.
**Table S3:** Changes in the intakes of sweets and snacks by category after 3 months of oral semaglutide treatment.

## Data Availability

The datasets generated during this study include personal health information and are not publicly available due to privacy and ethical considerations. Aggregated or anonymized data may be made available upon reasonable request by the corresponding author, subject to institutional and ethical approvals.
