# Supplementary material for: Effects of Oral Semaglutide on Dietary Intake and Body Composition in Japanese People With Type 2 Diabetes: A Prospective Observational Study in Clinical Practice
Source: Endocrinol Diabetes Metab. 2026 Aug 4;9(5):e70295. doi: 10.1002/edm2.70295 (PMC13437943; doi:10.1002/edm2.70295)
Supplement: Supplementary file 1 [file EDM2-9-e70295-s001.docx]

**Supplemental Table 1. Daily step count distribution at baseline and after 3 months of oral semaglutide treatment**

| Daily step count  （steps/day） | Baseline  (n=25) | After 3 months (n=25) | *p-value* |
| --- | --- | --- | --- |
| < 3000 | 8 (32.0) | 12 (48.0) | 0.518 |
| 3000–6000 | 8 (32.0) | 4 (16.0) |  |
| 6000–8000 | 3 (12.0) | 3 (12.0) |  |
| 8000–10,000 | 3 (12.0) | 2 (8.0) |  |
| > 10,000 | 3 (12.0) | 4 (16.0) |  |

Values are presented as n (%). The p-value for the change in daily step count category between baseline and the end of 3 months of oral semaglutide treatment was calculated using the Wilcoxon signed-rank test. *p* < 0.05 was considered as being indicative of statistical significance.

**Supplemental Table 2. Individual changes in the daily step count category after 3 months of oral semaglutide treatment**

| Change in daily step count category | n (%) |
| --- | --- |
| Increased | 2 (8.0) |
| Unchanged | 19 (76.0) |
| Decreased | 4 (16.0) |

The number of participants in whom the daily step count category increased, remained unchanged, or decreased from baseline to the end of 3 months of oral semaglutide treatment are presented as n (%).

**Supplemental Table 3. Changes in the intakes of sweets and snacks by category after 3 months of oral semaglutide treatment**

|  | **All (n=25)** | | | **Male (n=12)** | | | **Female (n=13)** | | |
| --- | --- | --- | --- | --- | --- | --- | --- | --- | --- |
| **Categories of sweets and snacks** | **Baseline** | **After 3 months** | **p-value** | **Baseline** | **After 3 months** | **p-value** | **Baseline** | **After 3 months** | **p-value** |
| a. Japanese sweets (wagashi) | 0.9 ± 0.2 | 0.4 ± 0.2 | **<0.05***^‡^ | 0.4 ± 0.3 | 0.4 ± 0.3 | 1.000 | 1.3 ± 0.4 | 0.4 ± 0.2 | **<0.05***^†^ |
| b. Sweet breads and cakes | 1.3 ± 0.3 | 0.5 ± 0.2 | **<0.01****^‡^ | 0.6 ± 0.3 | 0.0 ± 0.0 | 0.066 | 1.9 ± 0.4 | 1.0 ± 0.3 | **<0.05***^‡^ |
| c. Fried snack foods | 0.9 ± 0.4 | 0.2 ± 0.1 | **<0.01****^‡^ | 1.2 ± 0.7 | 0.3 ± 0.2 | 0.068 | 0.6 ± 0.2 | 0.2 ± 0.1 | **<0.05***^‡^ |
| d. Rice crackers and cookies | 1.4 ± 0.4 | 0.8 ± 0.3 | 0.080 | 0.8 ± 0.3 | 0.5 ± 0.2 | 0.336 | 1.9 ± 0.7 | 1.0 ± 0.6 | 0.173 |
| e. Ice cream | 1.3 ± 0.4 | 0.6 ± 0.2 | 0.205 | 0.8 ± 0.4 | 0.5 ± 0.3 | 0.171 | 1.8 ± 0.8 | 0.7 ± 0.3 | 0.205 |
| f. Chocolate | 0.9 ± 0.2 | 0.8 ± 0.3 | 0.485 | 0.9 ± 0.4 | 0.3 ± 0.2 | **<0.05***^‡^ | 0.8 ± 0.3 | 1.2 ± 0.6 | 0.576 |
| g. Candies | 0.5 ± 0.2 | 0.6 ± 0.4 | 0.671 | 0.3 ± 0.2 | 0.1 ± 0.1 | 0.414 | 0.8 ± 0.5 | 1.0 ± 0.8 | 1.000 |
| h. Jelly and pudding | 0.5 ± 0.2 | 0.4 ± 0.2 | 0.150 | 0.3 ± 0.3 | 0.2 ± 0.1 | 0.655 | 0.7 ± 0.3 | 0.7 ± 0.3 | 1.000 |
| Sweet foods that require chewing ^§^ (a, b, c, d) | 4.4 ± 0.8 | 1.9 ± 0.4 | **<0.01****^‡^ | 3.0 ± 1.2 | 1.2 ± 0.5 | **<0.05***^‡^ | 5.7 ± 1.0 | 2.6 ± 0.7 | **<0.05***^‡^ |
| Sweet foods that require little or no chewing ^§^ (e, f, g, h) | 3.2 ± 0.7 | 2.4 ± 0.6 | 0.252 | 2.2 ± 0.7 | 1.1 ± 0.3 | 0.159 | 4.1 ± 1.1 | 3.6 ± 1.0 | 0.875 |

Intake of sweets and snacks was evaluated using predefined portion-based categories. Values are presented as means ± SE. *p*-values are calculated using paired t-tests for normally distributed variables and the Wilcoxon signed-rank test for non-normally distributed variables. ^†^Paired t-test; ^‡^Wilcoxon’s signed-rank test. ^§^ Sweet foods were classified according to the chewing requirement: Items a-d represent sweet foods that require chewing, while items e-h represent foods that require little or no chewing.***p* < 0.01; **p* < 0.05. *p*-values shown in bold indicate statistically significant differences.
